# Supplementary material for: Harnessing protein folding neural networks for peptide–protein docking
Source: Nat Commun. 2022 Jan 10;13:176. doi: 10.1038/s41467-021-27838-9 (PMC8748686; doi:10.1038/s41467-021-27838-9)
Supplement: Supplementary file 1 — Supplementary Information [file 41467_2021_27838_MOESM1_ESM.pdf]

Harnessing protein folding neural networks for peptide-protein docking

Tomer Tsaban[1,#], Julia K. Varga[1,#], Orly Avraham[1,#], Ziv Ben-Aharon[1], Alisa Khramushin[1], and Ora Schueler-Furman[1,*]

[1]Department of Microbiology and Molecular Genetics, Institute for Biomedical Research Israel-Canada, Faculty of Medicine, The Hebrew University of Jerusalem, Jerusalem, Israel

[#] These authors contributed equally to this work
* Correspondence should be addressed to O.S.F. (ora.furman-schueler@mail.huji.ac.il)

– Supplementary Information –

Supplementary Figures

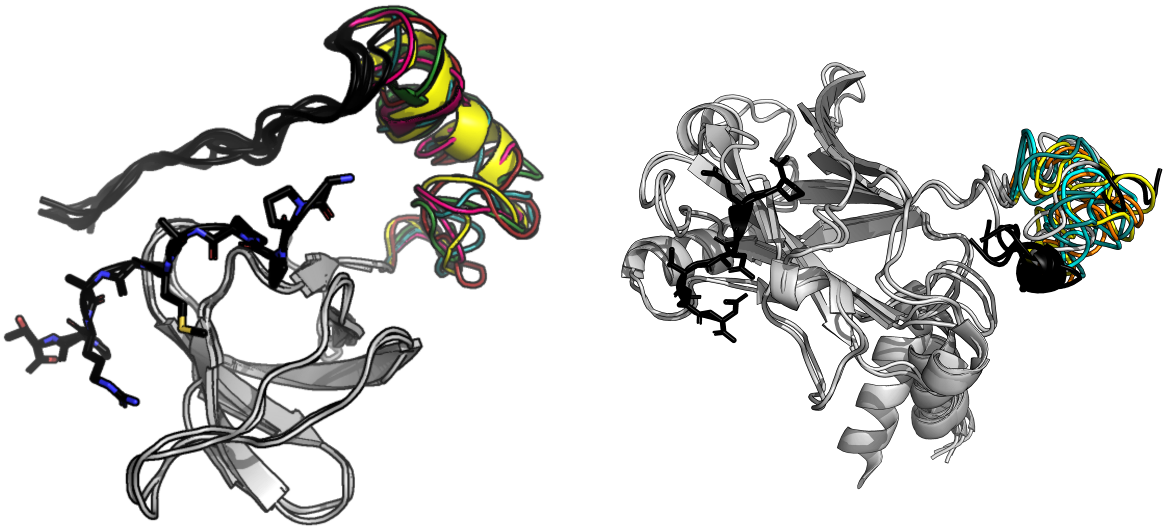

Supplementary Figure 1: RoseTTAFold attempts to fold the polyglycine linker. (accompanies Figure 1A)

Peptide-protein complexes PDB IDs 1ssh[1] (left) and 1czy[2] (right) modeled with RoseTTAFold. The receptor structures are modeled well (light gray, all aligned to the crystal structure), yet the peptides do not reach the binding site (peptides are in black, the peptide from the crystal structure shown in sticks). The polyglycine linker (30 glycines) is folded into an alpha-helix (left) or a highly disordered globule (right), none of which is appropriate for completing the task of peptide docking. AF2 treats polyglycine differently (compared to the AF2 predictions shown in Figure 1A).

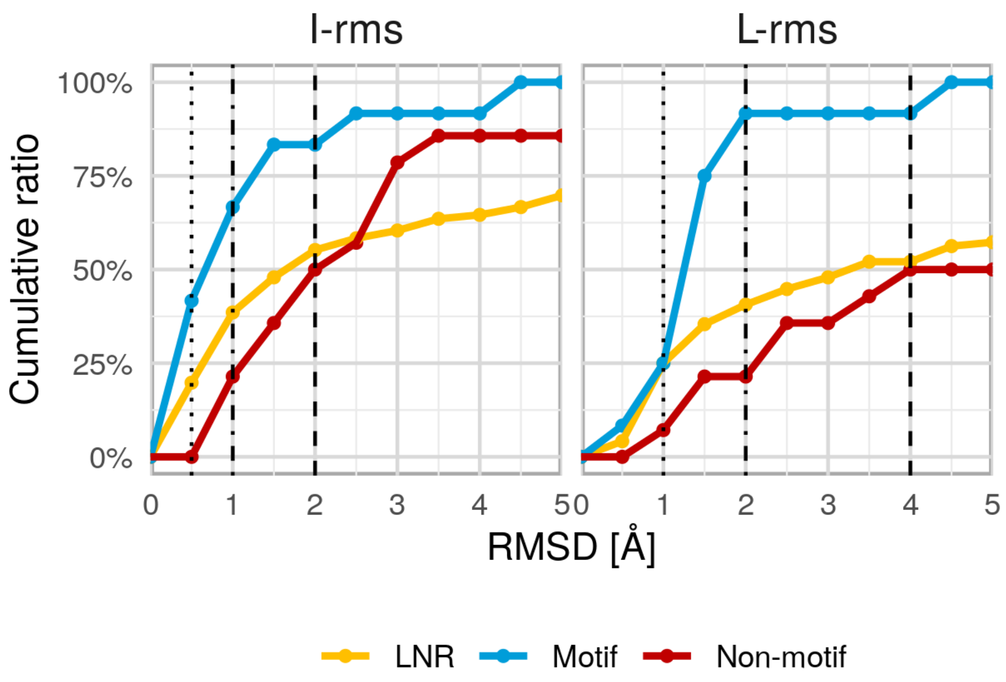

Supplementary Figure 2: Results of AF2 peptide docking performance, according to CAPR Imeasures (accompanies Figure 1B). Cumulative plots are shown for (A) Interface RMSD ,I-RMS, calculated over the interface after its alignment (same as Figure 1B, right upper plot). (B)Ligand RMSD, L-RMS, calculated over the peptide after receptor alignment. Cutoffs used i nCAPRI to define the quality of models are highlighted: acceptable - medium - high quali tymodels are defined for the ranges of 2.0,1.0, and 0.5 Å for I-RMSD, and 4.0, 2.0 and 1.0 Å f orL-RMSD. Source data are provided as a Source Data file.

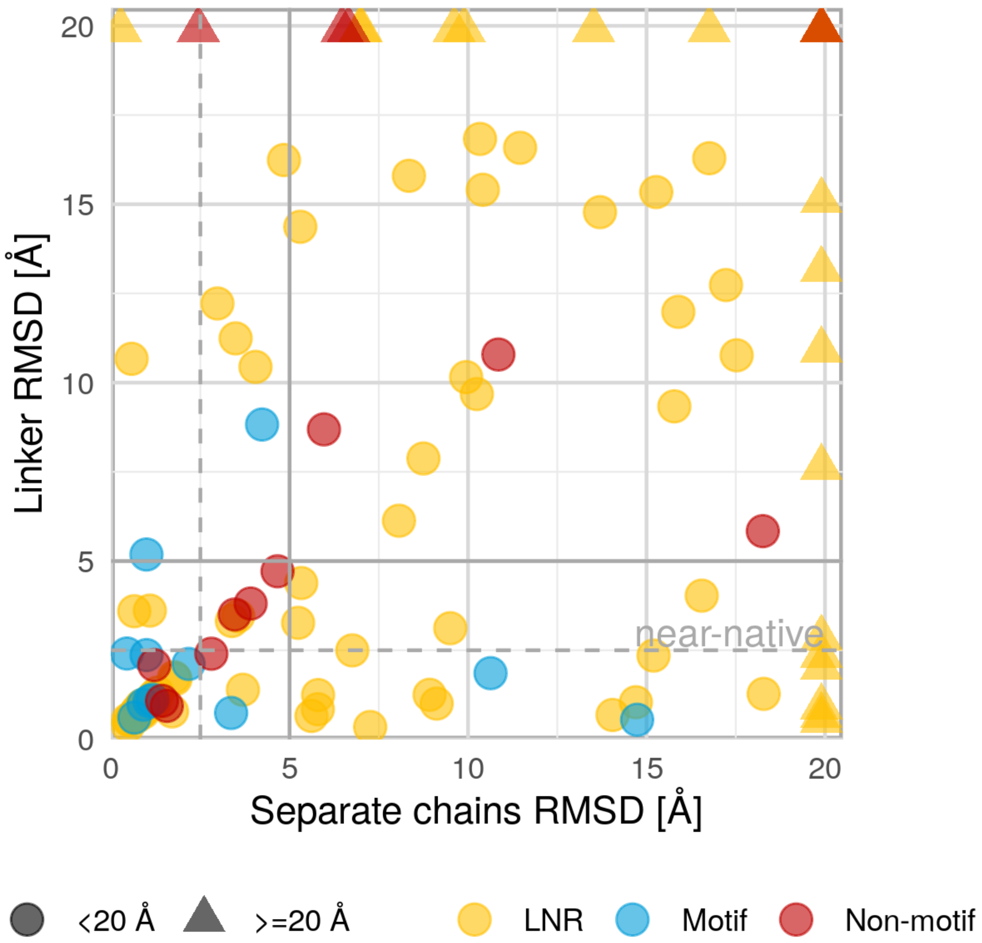

Supplementary Figure 3: Complementary performance of AF2 peptide docking using two different implementations (accompanies Figure 1B). Shown are results for peptides connected to the receptor via a polyglycine linker (y-axis) vs. peptides provided as separate chain (x-axis) .Based on these results, we decided to assess performance based on the best model result fro mboth implementations (total of 5+5=10 models).
Source data are provided as a Source Data file.

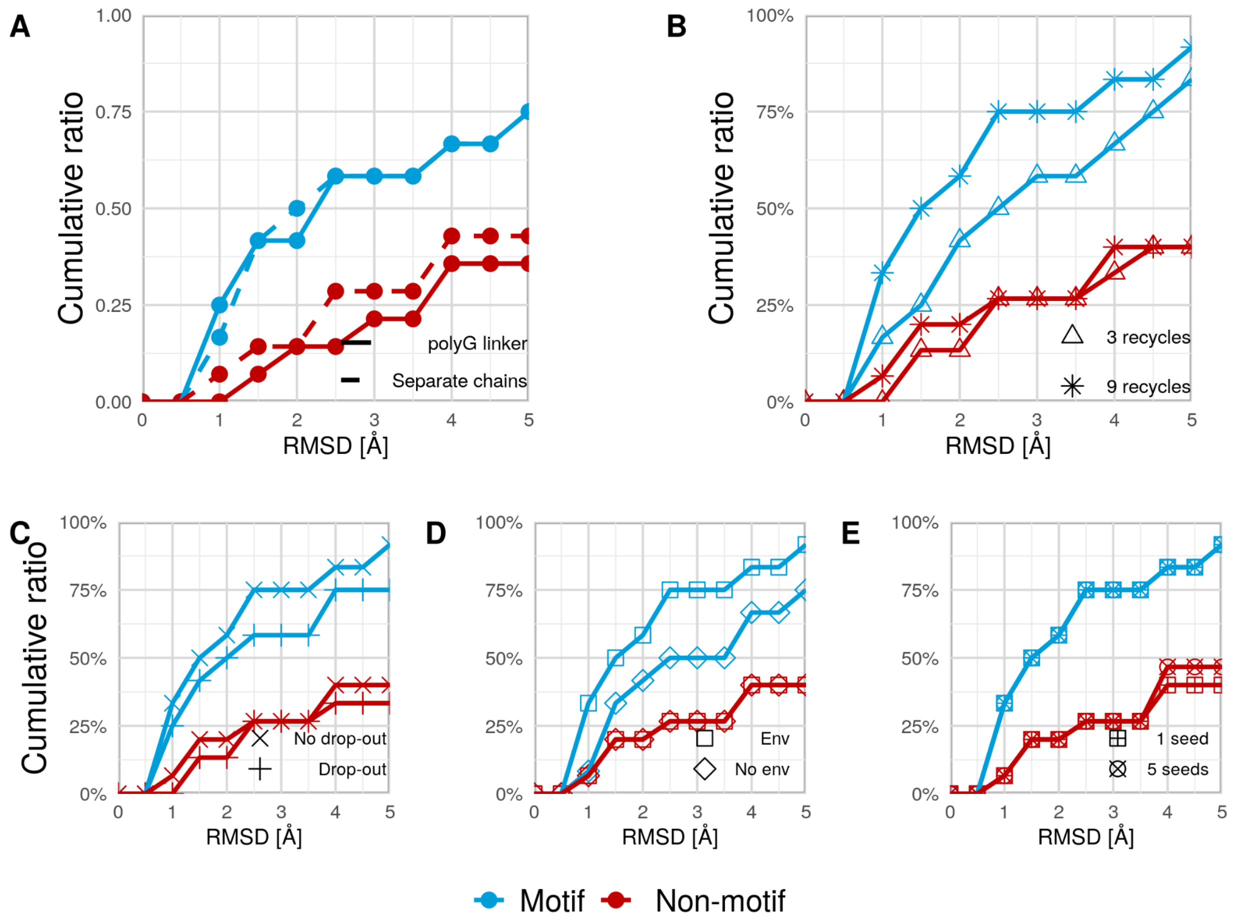

Supplementary Figure 4: Optimization of parameters for AF2 peptide docking (accompanies Figure 1B). Shown is cumulative performance for variations of a set of parameters. On eparameter is varied in each plot, in the background of the final setup (i.e., polyG linker +separate chains; 9 cycles; no dropout; inclusion of environmental sequences in the MS Asimulations; 1 random seed). (A) Best performance is obtained when combining runs wi thpolyglycine linkers and separate chains. (B) Increasing the number of cycles from 3 to 9improves performance. (C) Dropout reduces performance for the motif set. (D) Inclusion ofenvironmental sequences does not affect performance. (E) Addition of seeds does not impro veperformance. Source data are provided as a Source Data file.

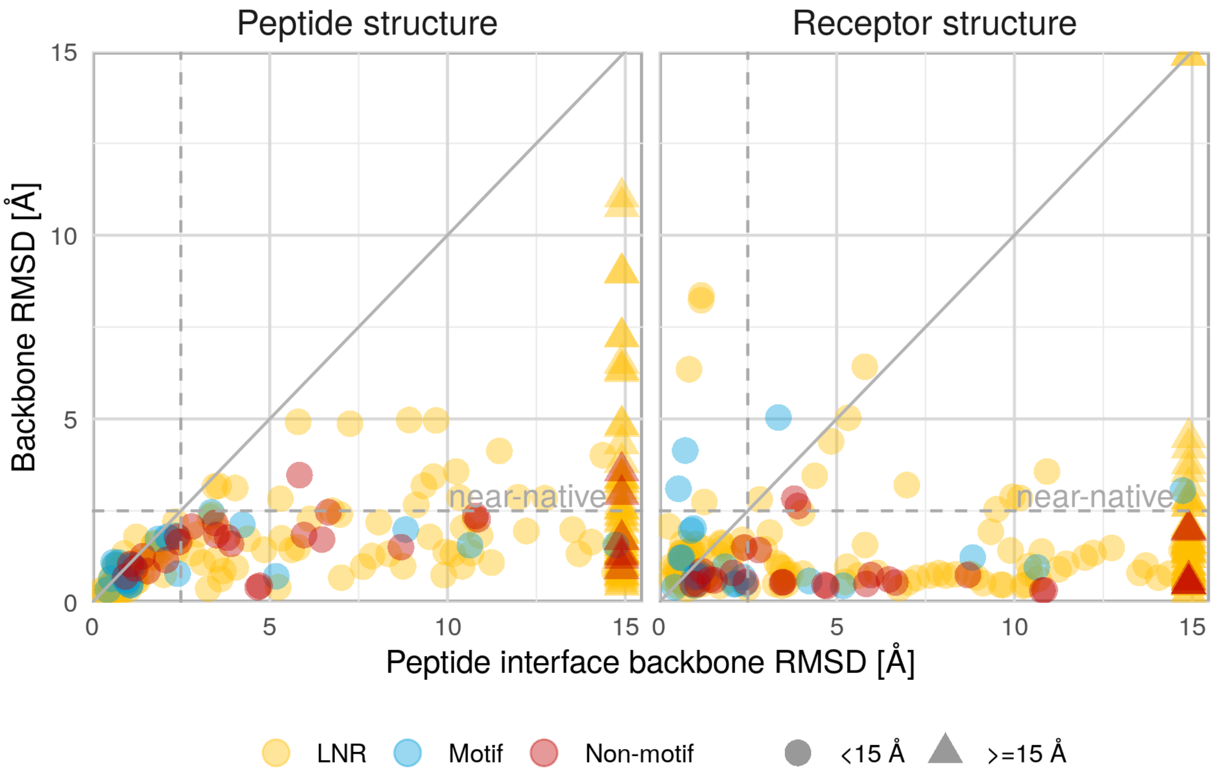

Supplementary Figure 5: Accurate modeling of the peptide structure (left) or receptor structure (right) does not guarantee accurate modeling of the interface (accompanies Figure 1D). Correlation between peptide interface backbone RMSD and receptor accuracy or peptid eaccuracy in AF2 models. Source data are provided as a Source Data file.

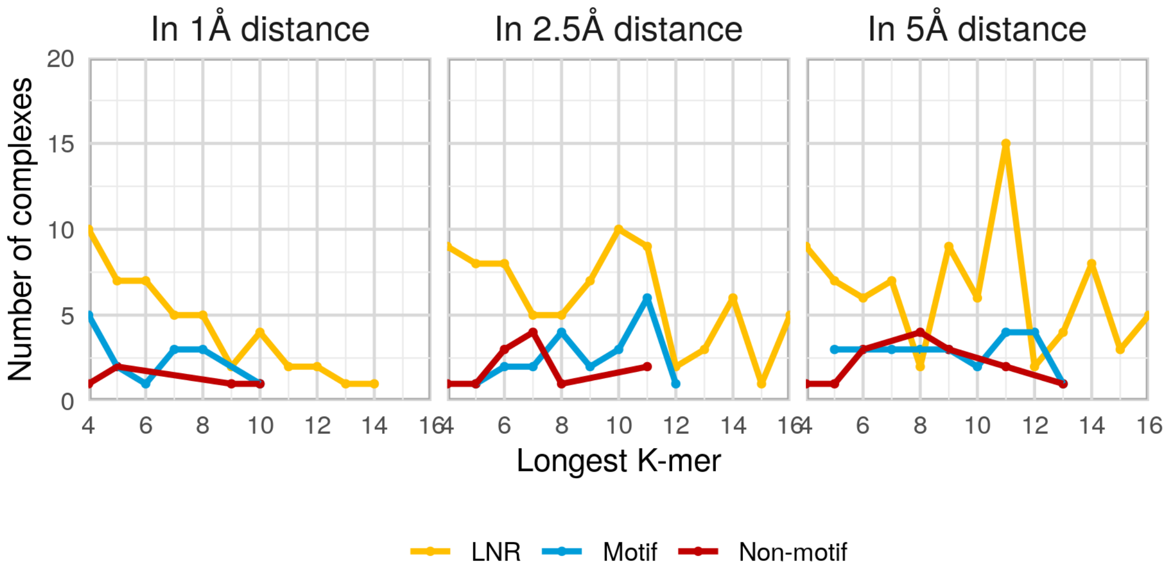

Supplementary Figure 6. Long consecutive peptide stretches that are accurately modeled ar egood candidates for further study of possible binding motifs (accompanies Figure 2). Shown ar ethe counts of longest consecutive stretches of well predicted residues, within 1,2.5 and 5 Åpeptide interface residue backbone distance, respectively.
Source data are provided as a Source Data file.

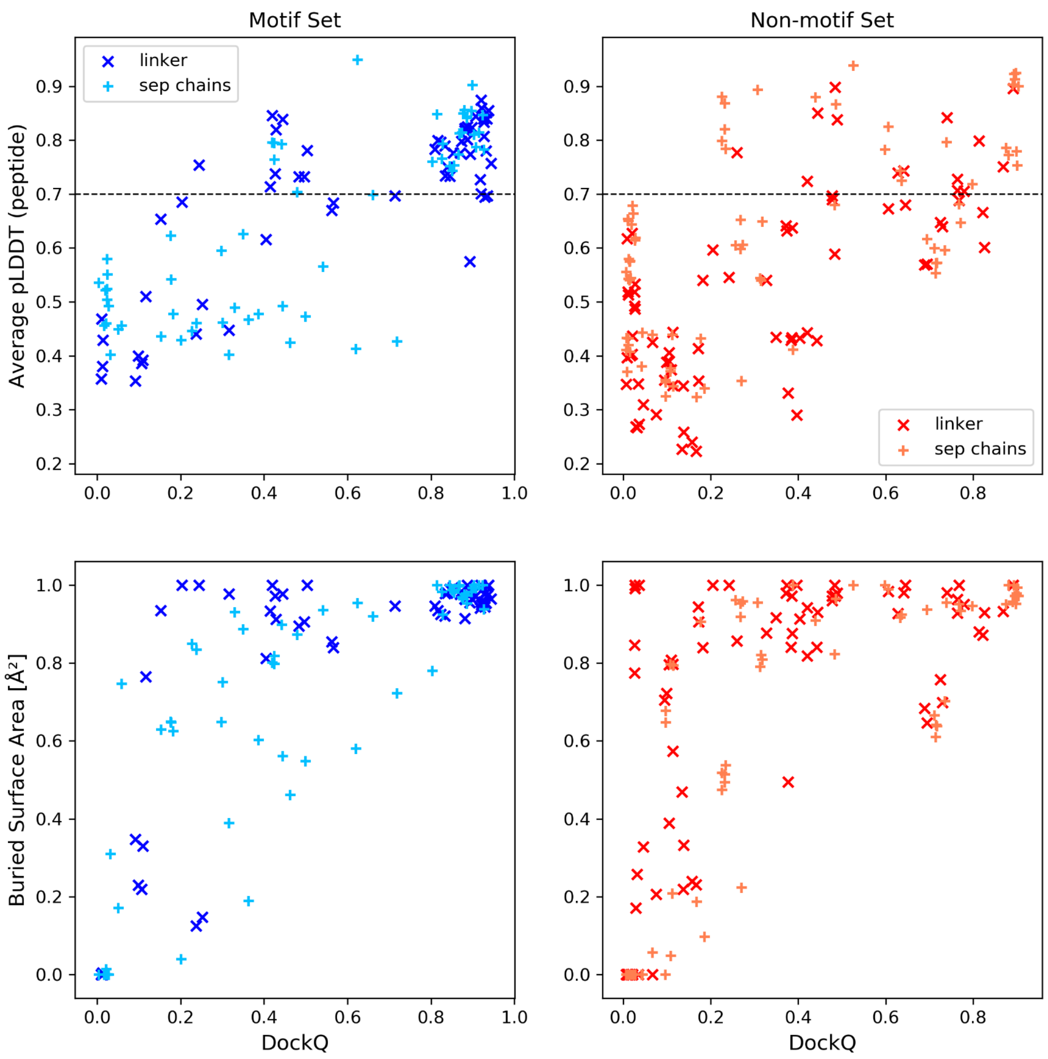

Supplementary Figure 7. Separation of accurate from inaccurate models using pLDDT and buried surface area. Left column: The motif set (in shades of blue). Right column: The non-moti fset (in shades of red). Upper panel: Average pLDDT (of peptide) *vs.* DockQ metric. Lowe rpanel: Interface buried surface area (normalized to the model with maximal value for each pdb)*vs.* DockQ metric. Each mark represents one model (5 with linker and 5 without, 10 total fo reach pdb). Models with linker are represented by dark colored “x” and no linker (separat echains) by pale “+”. Source data are provided as a Source Data file.

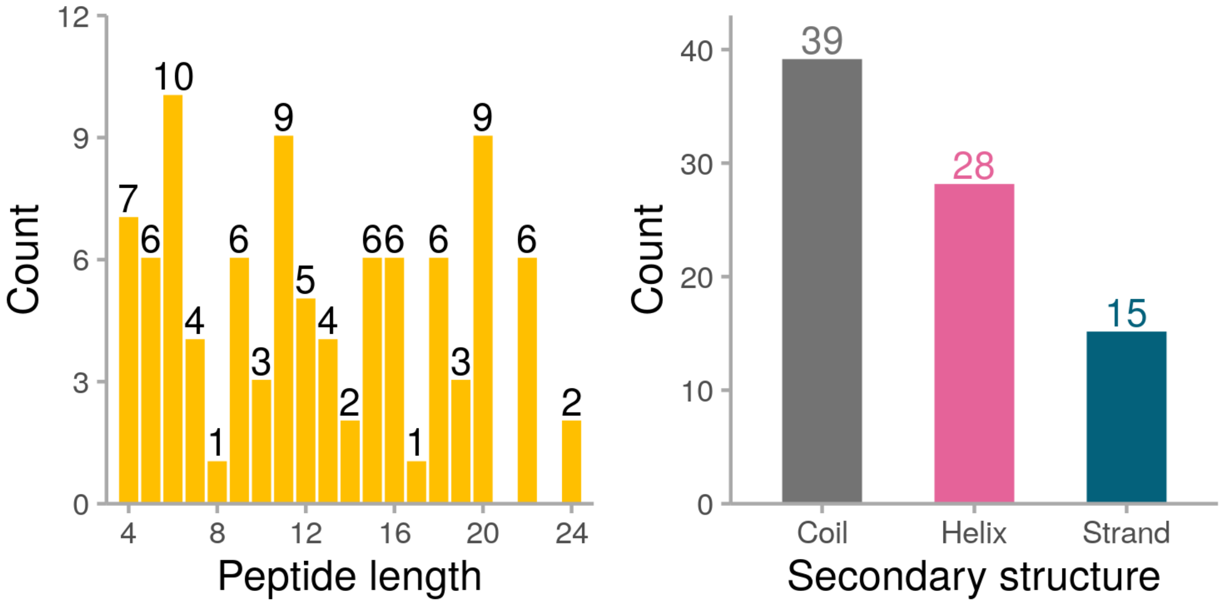

Supplementary Figure 8. Characteristics of the LNR dataset. Distributions are shown for peptid elength (left), and secondary structure (right). Source data are provided as a Source Data file.

Supplementary References

1. Kursula, P., Kursula, I., Lehmann, F., Song, Y.-H., Wilmanns, M. 1SSH: Crystal structure of the SH3 domain from a S. cerevisiae hypothetical 40.4 kDa protein in complex with a peptide. http://doi.org/10.2210/pdb1SSH/pdb
2. Ye, H., Park, Y. C., Kreishman, M., Kieff, E., & Wu, H. The structural basis for the recognition of diverse receptor sequences by TRAF2. *Molecular cell*, *4*(3), 321-330 (1999).
