# Supplementary material for: Harnessing protein folding neural networks for peptide–protein docking
Source: Nat Commun. 2022 Jan 10;13:176. doi: 10.1038/s41467-021-27838-9 (PMC8748686; doi:10.1038/s41467-021-27838-9)
Supplement: Supplementary file 3 — Description of additional supplementary files [file 41467_2021_27838_MOESM3_ESM.docx]

File name: Supplementary Data 1

Description: Datasets of structures of peptide-protein interactions used in this study, including AF2 modeling performance using a peptide connected to the receptor by a poly-glycine linker, or a peptide submitted as a separate chain.

File name: Supplementary Data 2

Description: Detection of interface hotspot residues on AF2 models. For different sets and partners, we report Spearman’s rank correlation and prediction success (based on hotspot definition using ΔΔG = 1.5 kcal/mol as threshold). Note how the fraction of True Positives among all Positives (hotspots) increases dramatically when only accurate models (within 2.5Å RMSD) are inspected - reducing significantly the False Negative rate.

File name: Supplementary Data 3

Description: Effect of possible presence of monomer templates covering the peptide-protein interaction on AF2 prediction accuracy. Included are the PDB ID-s from the 3 datasets, with their corresponding fusion equivalents. For all pairs, it is indicated if the fusion and the native of the modeled complex have identical interfaces as well as the best rmsBB_if for the complex modeled with AF2.

File name: Supplementary Data 4

Description: Effect of providing the native complex structure as input to AF2 on modeling accuracy. All RMS values are rmsBB_if, each column shows the values for specific AF2 models, e.g., best_by_models_345 is the best rmsBB_if value among the models produced by networks 3-5. For this comparison, all models were created using separate chains.

File name: Supplementary Data 5

Description: List of PDB entries used in this study.
