# Supplementary material for: Harnessing protein folding neural networks for peptide–protein docking
Source: Nat Commun. 2022 Jan 10;13:176. doi: 10.1038/s41467-021-27838-9 (PMC8748686; doi:10.1038/s41467-021-27838-9)
Supplement: Supplementary file 9 — Reporting Summary [file 41467_2021_27838_MOESM9_ESM.pdf]

nature portfolio

Corresponding author(s): Ora Schueler-Furman

Last updated by author(s): Dec 8, 2021

Reporting Summary

Nature Portfolio wishes to improve the reproducibility of the work that we publish. This form provides structure for consistency and transparency in reporting. For further information on Nature Portfolio policies, see our Editorial Policies and the Editorial Policy Checklist.

Statistics

For all statistical analyses, confirm that the following items are present in the figure legend, table legend, main text, or Methods section.

n/a	Confirmed	
☐	☒	The exact sample size (*n*) for each experimental group/condition, given as a discrete number and unit of measurement
☒	☐	A statement on whether measurements were taken from distinct samples or whether the same sample was measured repeatedly
☐	☒	The statistical test(s) used AND whether they are one- or two-sided<br>*Only common tests should be described solely by name; describe more complex techniques in the Methods section.*
☐	☒	A description of all covariates tested
☒	☐	A description of any assumptions or corrections, such as tests of normality and adjustment for multiple comparisons
☐	☒	A full description of the statistical parameters including central tendency (e.g. means) or other basic estimates (e.g. regression coefficient) AND variation (e.g. standard deviation) or associated estimates of uncertainty (e.g. confidence intervals)
☒	☐	For null hypothesis testing, the test statistic (e.g. *F*, *t*, *r*) with confidence intervals, effect sizes, degrees of freedom and *P* value noted<br>*Give P values as exact values whenever suitable.*
☒	☐	For Bayesian analysis, information on the choice of priors and Markov chain Monte Carlo settings
☒	☐	For hierarchical and complex designs, identification of the appropriate level for tests and full reporting of outcomes
☐	☒	Estimates of effect sizes (e.g. Cohen's *d*, Pearson's *r*), indicating how they were calculated

Our web collection on statistics for biologists contains articles on many of the points above.

Software and code

Policy information about availability of computer code

Data collection	All code and data used in this study are open source and available (through reference); all custom code written for this work and the data generated correspondingly, is available through a minted github repository at https://github.com/Furman-Lab/Peptide_docking_with_AF2_and_RosettAfold
Data analysis	All code and data used in this study are open source and available (through reference); all custom code written for this work and the data generated correspondingly, is available through a minted github repository at https://github.com/Furman-Lab/Peptide_docking_with_AF2_and_RosettAfold

For manuscripts utilizing custom algorithms or software that are central to the research but not yet described in published literature, software must be made available to editors and reviewers. We strongly encourage code deposition in a community repository (e.g. GitHub). See the Nature Portfolio guidelines for submitting code & software for further information.

Data

Policy information about availability of data

All manuscripts must include a data availability statement. This statement should provide the following information, where applicable:

- Accession codes, unique identifiers, or web links for publicly available datasets
- A description of any restrictions on data availability
- For clinical datasets or third party data, please ensure that the statement adheres to our policy

All source data are provided with this paper. These data, as well as models generated in this study, are available at:
https://github.com/Furman-Lab/Peptide_docking_with_AF2_and_RosettAfold
PDB entries used in this study are fully listed in the data availability statement in the manuscript.

Field-specific reporting

Please select the one below that is the best fit for your research. If you are not sure, read the appropriate sections before making your selection.

- [x] Life sciences
- [ ] Behavioural & social sciences
- [ ] Ecological, evolutionary & environmental sciences

For a reference copy of the document with all sections, see nature.com/documents/nr-reporting-summary-flat.pdf

Life sciences study design

All studies must disclose on these points even when the disclosure is negative.

Sample size	Sample size was not "chosen" but is a result of our data filtering process, according to field standards, detailed extensively in the methods and previous works by us and others. We use a small, previously published dataset for calibration and a larger one, generated for this work, for validation and assessment.
Data exclusions	No data was excluded
Replication	Replications are not relevant to this study. The described protocols were optimized and observed to converge. Nonetheless we experimented with stochastic parameters, which had little influence on the results, as described in the manuscript.
Randomization	Sampled-based group randomization is not relevant to this study. Predicted models were compared against ground truth as the standard procedure for such a protocol.
Blinding	Blinding is not relevant to this study, as we compare a computational prediction to ground truth data, and no dedicated "machine" training was performed (hence, no need for a "hold out" set).

Reporting for specific materials, systems and methods

We require information from authors about some types of materials, experimental systems and methods used in many studies. Here, indicate whether each material, system or method listed is relevant to your study. If you are not sure if a list item applies to your research, read the appropriate section before selecting a response.

Materials & experimental systems

n/a	Involved in the study
[x]	[ ] Antibodies
[x]	[ ] Eukaryotic cell lines
[x]	[ ] Palaeontology and archaeology
[x]	[ ] Animals and other organisms
[x]	[ ] Human research participants
[x]	[ ] Clinical data
[x]	[ ] Dual use research of concern

Methods

n/a	Involved in the study
[x]	[ ] ChIP-seq
[x]	[ ] Flow cytometry
[x]	[ ] MRI-based neuroimaging
